# Implementing Aging in Place in Hong Kong: Meeting the Needs and Aspirations of Older Adults and Their Caregivers Living in Private Housing

**DOI:** 10.3390/ijerph21030348

**Published:** 2024-03-14

**Authors:** Jean Woo, Keilee Mok, Wui-Ling Chu, Regina Lo, Rina Ko

**Affiliations:** 1 Jockey Club Institute of Ageing, The Chinese University of Hong Kong, Hong Kong SAR, China; wuilingchu@cuhk.edu.hk (W.-L.C.); reginayhlo@cuhk.edu.hk (R.L.); rinako@cuhk.edu.hk (R.K.); 2 Department of Medicine & Therapeutics, Faculty of Medicine, The Chinese University of Hong Kong, Hong Kong SAR, China; 3 Kreative Beans, Hong Kong SAR, China; kmkreativebeans@outlook.com

**Keywords:** aging in place, healthy aging, older adults, carer

## Abstract

A policy of aging in place should be accompanied by physical and social environments that support healthy aging. This article describes how a property development company in Hong Kong sought to elicit the views of older people and their caregivers towards elderly services through a market research company, using questionnaire surveys followed by focus groups. Over 80% of all participants rated healthy dietary habits and exercise, maintaining mental and spiritual health, and maintaining a generally healthy lifestyle as important. Current health concerns include long waiting times for care at public hospitals, lack of carer should dependency occur, and lack of information about what services are available in the community. Interests in services in their neighbourhood include medical care (82%), healthy lifestyle activities (66%), and home care support (55%). There was considerable interest in the provision of services that improve brain and physical function, as well as general health checks. Carers were willing to pay more for services compared with older adults themselves. The findings inform the development of pilot models of aging in place as a sustainable financial model.

## 1. Introduction

Hong Kong has an aging population, with 1.45 million people aged 65 years and over (19.6% of the total population), of which 590,000 live in private domestic households (Population Census 2021 https://www.census2021.gov.hk/en/index.html) accessed on 24 January 2024. The government has articulated a policy of aging in place, supported by residential care only if necessary. Furthermore, the private sector is being encouraged to provide services for older adults [1]. Older adults also expressed a wish to remain in the same residence and community which they are familiar with, rather than move to residential care [2]. However, aging in place must be accompanied by physical and social surroundings that enable healthy aging as defined by the World Health Organization; that is, the ability to optimize and maintain functioning (both cognitive and physical) in the context of an age-friendly neighbourhood [3]. There is a paucity of data from the perspective of aging adults regarding aging in place. Previously reported interaction with older adults suggested that safety, spacious and comfortable environment, age-friendly facilities, meeting their varied needs, caring support, and home maintenance are valued characteristics [4]. At the same time, there is a rapidly evolving senior living landscape where property development companies and some non-government organizations design purpose-built residences to suit older adults from varying socioeconomic groups (Ventria Residence (www.ventriaresidence.com) accessed on 24 January 2024, Tanner Hill (www.thetannerhill.hkhs.com) accessed on 24 January 2024, Blissful Place (blissfulplace.hkhs.com) accessed on 24 January 2024, Cheerful Court (sen.hkhs.com/projects/cheerful-court?id=about-project) accessed on 24 January 2024 and Jolly Place (sen.hkhs.com/projects/jolly-place?id=about-project) accessed on 24 January 2024). Due to the high density living in Hong Kong, the majority of people live in high-rise blocks of flats supported by property management companies. There may be common areas within a block of flats suitable for various community activities, or open spaces/gardens within housing complexes shared by a group of high-rise buildings. This existing infrastructure may form a base from which various services and health-promoting activities may be developed to meet the needs and aspirations of older adults who do not wish to move from their current residences. Aging in place involves more than the hardware of building design, including social networks and available services to meet needs, as well as activities to promote healthy aging that takes into account retarding the aging process itself and not just targeting chronic disease prevention and management.

Several themes related to aging in place have been highlighted recently. These studies show that a holistic landscape involving many disciplines and services is needed, and they need to be integrated with the older adult in the centre. The concept of a socially active neighbourhood has been proposed to support aging in place [5]. Remote consultations [6], empowerment of caregivers [7], lifestyle redesign [8], mobility training for those who are frail [9], early identification and management of mental health [10], community exercise programmes [11], and programmes designed following a comprehensive assessment of needs [12,13] have been discussed. In particular, poor mobility capacity has been identified as a potent modifiable risk factor for incident disability and the quality of life [14]. This is particularly relevant in light of the finding that increasing longevity in Hong Kong has not been accompanied by the same increase in disability-free life expectancy [15]. In other words, there is an increasing burden of disability, particularly for women. Technology has been featured prominently in the design of senior living. However, a recent review suggests that smart living environments based on unobtrusive technologies had low-to-moderate effectiveness to support older adults to age in place, and that such technologies are not mature enough for widespread adoption [16]. Some communities have developed aging in place models that include different categories of services: daily living needs, physical and mental health needs, and self-fulfilment needs such as continuous learning, leisure, etc. [17].

How these principles may be implemented in Hong Kong would involve multiple sectors: the government (mainly by formulating clearcut policies and supporting service providers), the private and non-government organizations. There may be different models for different socioeconomic groups; however, the basic components should be universal: to embrace the health equity principle that aging in place should not be enjoyed by those who can afford it. Another important principle is that older adults themselves should express their needs for aging in place to architects and building designers, management companies, local health and social services network, as well as promoters of healthy aging. We explored the development of such a model for private housing, based on a quantitative survey of residents followed by focus groups living in the New Territories East Region of Hong Kong. These were carried out by a consultant market research company commissioned by a prominent private property development company in Hong Kong, that is interested in the development of new models for senior living.

## 2. Materials and Methods

A mixed-method design (consisting of both a questionnaire survey followed by focus groups) was used by a market research company commissioned by a private property developer to elicit the views of older people and their caregivers towards elderly services that are currently in place, and the development of desirable newer services. The quantitative part of the study involved a questionnaire, collecting responses regarding their interest and experiences towards elderly services provided in their community. This was followed by conducting focus group interviews, where questionnaire topics were discussed in greater depth with the aim to capture a more detailed experience of elderly services and their interest in desirable newer elderly services.

### 2.1. Participants of the Questionnaire

The inclusion criteria consisted of participants being aged 30 years or above, with participants aged 30 to 40 years having to be a caregiver for an elderly aged 50 years or above. Participants were all residents of the selected Hong Kong New Territories East Region properties owned by the property company that commissioned the study, as well as residents of other private estates, and did not have family members working in sensitive areas (e.g., marketing research, commercial, publicity, media, and real estate). They were approached through either mail surveys or random street encounters. Participation was voluntary, and participants were divided into two groups: the elderly and the caregiver group, and questions differed according to participant groupings. Participants in the caregiver group were asked about what they think care recipients need, while older people responded according to their own perspectives. The questionnaire covered information about education, household income, living circumstance, self-rated health, number of chronic diseases, health goals, unmet needs, awareness of current elderly services, and service access. Their willingness to pay for the development of new services as well as preferred communication channels were also explored. The Chi square test was used to examine statistical difference between genders, with a *p* value of <0.05 regarded as statistically significant.

### 2.2. Focus Group

Four focus groups consisting of 25 people were held: two for residents of the property company (one for older adults, and one for caregivers), and two for residents of the other private estates. There were 6–7 people in each group, with each session lasting about two hours. The participants were divided into three distinct subgroups. The first group consisted of those aged 50–65 years, many of whom were still working. Some had just retired or were housewives. They had more active lifestyles and were prepared to spend more on health maintenance and services compared to the other groups. One group consisted of older people who were aged 65 years or above, who had adjusted to life in retirement. Although they had a few chronic diseases such as hypertension, they did not have any mobility limitations. They tended to have adopted a more regular lifestyle. The third group consisted of people aged 30–50 years, taking care of older family members who are mostly mobile, retired, or still working. These carers were all working full-time. The interviews were recorded for a detailed thematic analysis with illustrative quotes. For each group, all participants were encouraged to give their opinions on the topics being discussed. The recordings were then analysed, and the comments were grouped into themes and presented according to each of the distinct subgroups.

## 3. Results

### 3.1. Results from the Survey

The characteristics of the respondents are showed in Table 1.

The majority of respondents lived with a spouse or family, while approximately 2% lived alone. There were significantly more women in the younger age groups (<70 years), and more women who were carers. More men lived with a helper rather than with relatives or a spouse compared with women. Over 60% of the respondents rated their health as good, although 86% had up to two and 7–8% had three or more chronic diseases.

With reference to health goals, over 80% of all participants rated healthy dietary habits and exercise, maintaining mental and spiritual health, and maintaining a generally healthy lifestyle as important. The prevention of disease, having a good social network, and prevention of home accidents were rated as important goals by fewer people (76%, 48%, and 29%, respectively). Regarding unmet healthcare needs, at least half of the participants worried about long waiting times at public hospitals. Other major concerns included lack of a carer if the need arises and not enough money to pay for treatment. Concerns regarding affordability for treatment was more prevalent in the group with a household income of <40 K (HKD) compared to higher-income groups. Other concerns included a lack of information about what services are available in the community, especially 24 h emergency services. Over 70% of the participants adopted a healthy lifestyle as the major strategy for health management, with only approximately 30% making use of medical services, including Chinese medicine. Only 1% used smart health technology. In contrast, awareness of elderly services in the neighbourhood was the highest for medical consultations (60%) and much lower for supporting services (such as cleaning, personal care, safety alarm systems) (34%), and the lowest for healthy lifestyle-related services (27%).

Interest in services in their neighbourhood included medical care (82%), healthy lifestyle activities (66%), and home care support (55%). The desire for help with cleaning the home increased with the age of respondents, being the highest among those aged 70 years and above (12%). There was considerable interest in the provision of services that improve brain and physical function, as well as general health checks. The average amount participants were willing to pay monthly for such services was HKD 1441; carers were prepared to pay more compared with older adults themselves, while those with a monthly household income of HKD 70,000 and above were prepared to pay over HKD 2000. Of the few participants currently using elderly services (5%), the majority were satisfied with such services (83%). Over 60% of respondents expressed a wish for some sort of emergency support, particularly among those aged 70 years and over (68%). The majority relied on traditional media such as television, newspaper, friends, and relatives for information on elderly services (75% and 55%, respectively), with only 36% relying on information technology. Those with a higher household income tended to use online media more.

### 3.2. Results of the Focus Groups

The comments from the focus groups are described separately for three distinct groups of participants. The following are some themes, with illustrative quotes from different groups.

### 3.3. Comments from Older Adults (50–65 Years)

In general, participants in this group were still very much engaged with society through employment and did not regard themselves as “old”, viewing services for older people as not being for them. The comments show that they did not prepare for age-related declines in function and did not contribute much to the shaping of new services for aging in place.

“I am still employed, and it’s very busy at work. I am not sure what would happen when I retire.”

“I am currently semi-retired. I will find some work to do, exercise, or maybe so some part-time job.”

“I don’t like going to these community centres, only elderly would go there.”

“It’s called an elderly centre. Everyone who goes there are those who are very old… I won’t go there.”

### 3.4. Comments from Older Adults (>65 Years)

Retirement represented a life change, with both negative as well as positive connotations. There was little awareness about what services for older adults are available in their neighbourhood. Recent senior living housing catered to the very rich and not to them. They were also reluctant to spend large sums of money on major environmental modifications, preferring to save money instead. However, they appreciated a common area that is comfortable and well designed for social interaction and/or activities.

“I feel like I am very useless, and I don’t want to become a burden to my children.”

“I have been retired for many years. I was not used to it before. I have heard people say that I will die as soon as I retire… so I work as a volunteer now to help others.”

With regard to use of existing services for older adults:

“I rarely use these services, and I don’t know what other services there are. I only went to an elderly centre in City One Shatin after friends in my neighbourhood told me.”

“These elderly-targeted housing is for rich people, it does not target us.”

“It would be okay if it’s a small renovation such as for handrails and floor mats, as it won’t be that expensive. I won’t do it if it’s a whole renovation, it’s better to save some money.”

“I might not want to spend that much money, with not knowing how long I could keep using these things… I rather keep a bit more money with myself.”

“Having a common area in the estate could allow older people to get together for activities.”

“The most important thing is that it is well-renovated. If not, it would be like those community centres, which made me feel old. I won’t go there.”

“The atmosphere should have a common daily living flavour, so that we could sit down and chat comfortably.”

### 3.5. Caregiver

This group had the most comments about what older adults’ health and social needs are, as well as how the current services may be improved. Their views on what care recipients need seemed to be greater than those of the care recipients themselves, and they were willing to pay more. In general, the view was that the current government services did not meet the needs of older adults in maintaining health and social needs, while there was little information about self-financing services. Carers were willing to pay for home modification if necessary to enable independent functioning and were also prepared to consider major renovations. The choice to fit individual circumstances was important. Health-related activities on site for care recipients in a well-designed common space (as opposed to the unattractive current government-subverted centres) were desirable. Carers were concerned about the need for the prevention of functional decline and the need for such facilities to relieve carer burden.

“My father-in-law broke his left leg while playing football. His health is also worser after he recovered. These elderlies couldn’t bear these falls.”

“I am most concerned about his mental health after retirement. He seems to have nothing to do but sit around the house watching television all day.”

“If I renovate next time, I would like to do it all at once. So that I don’t have to worry about renovation when necessary.”

“The government’s services are not for us, we couldn’t access any of the services.”

“I really don’t know what companies in the market have these type of consulting services. Even if they do, it should be very expensive.”

“I really don’t know that there are these type of services on the market. It would be worth to pay more if it is not a hassle.”

“Other renovation companies have variable costs, it’s hard to say what is really necessary and what is not. It would be better if I could choose what I wanted.”

“The addition of handrails and anti-slipping are necessary as soon as possible. It’s better to pay a bit more to delay the risk of being in a wheelchair.”

“It would be nice if there are services such as physiotherapy and gym. Their children won’t have enough time to go with them every time.”

“As long as it is privately-owned and not thatdilapidated, it could already attract a lot of people.”

“It would allow them to have meaningful occupation to pass the time, so that they don’t have to stay at home and watch TV all day.”

## 4. Discussion

Among the residents living in private housing estates, similar concerns and aspirations relating to aging in place were shown in the survey and focus groups by older adults themselves, as well as carers. Although the majority had good health and mobility, they were concerned about maintaining health through the adoption of healthy lifestyles, rather than depending on medical services and chronic disease prevention through health checks as well as Chinese medicine consultations. Psychological health through interactions with friends and peers, participation in community activities through meaningful activities, and continued learning are features reflecting social needs and emphasizing self-value. At the same time, meeting future physical needs is important in terms of coping with possible physical dependencies, emergency care, access to healthcare professionals, safety of the living environment, as well as availability of activity programmes that maintain/promote psychological and physical health led by trainers. These aspirations closely match the action areas of the Decade of Healthy Aging promoted by the World Health Organization that includes creating an age-friendly environment and integrated primary care covering both social and medical elements. These aspirations differ from current health and social service providers’ views of what services need to be provided, as they are tackling health and social needs much further down the slope of decline in intrinsic capacity in the areas of frailty and disability, as well as a disease-based approach to prevention and management rather than the consequences of the aging process itself.

Another key point is that although there is information about public health and social services, not many people use it, probably because they cater to a slightly different profile of older people who are largely frail and dependent, or with multiple disabilities. Many social services are the means tested such that those with a household income above a certain low threshold will not be able to access them. There is also a perception that government services are for under-privileged groups and they do not wish to compete for services with those more in need. At the same time, there is little information relating to different service types and costs in the private sector that would meet the expressed needs of older adults and/or their carers. Information as well as services should ideally be provided in one place or centralized.

The information gathered in this market survey and focus groups provides important insights into the prerequisites for aging in place, enabling healthy aging to take place. Such information would form the basis for urban planning and estate management, to develop business models for services that could be provided on a fee-for-service basis, as well as promoting public preventive health in the community. For example, the presence of a care coordinator for an individual or groups of buildings could help develop services to match the needs expressed, as well as be the first point of contact for any needs from older persons or their carers. In this way, residents may be aware and access all existing and future health and social care resources in both public and private sectors through a trusted provider. Other than district health and their satellite centres, public and private medical clinics and hospitals navigating through various government voucher schemes for community health and social services, as well as relevant insurance schemes, could be developed. Home repair and re-modelling services could also be developed. A communal space could be designed or re-furbished to enable various group activities that are beneficial for maintaining cognitive as well as physical function, promote psychological well-being, and provide continuing learning.

The use of technological products would complement these models, such as self-assessment and educational apps linking self-evaluation to actionable activities, and relevant neighbourhood services [18,19] https://www.cadenza.hk/e-tools/en/ihealthscreen/ (accessed on 24 January 2024). Such an initiative may be implemented as a pilot in the private sector and the results evaluated. There is a potential for the model to be adopted by public housing estates or partially government subverted housing estates.

The findings from this survey and focus group support and inform the development of a Health In Place (HIP) initiative that is being considered by the private development company. The model emphasises preventive care, through the formation of a centralized hub for care coordination and the exchange of health information, with the aim of enhancing the overall well-being of the population. It prioritizes lifestyle wellness as a vehicle for improving general health, incorporating health education and regular health assessments to deepen individuals’ understanding of their health status and needs. Additionally, it provides health monitoring services as part of a broader strategy to foster a healthy and cohesive community environment.

There are several limitations to this study. It is essentially exploratory in nature, riding on the desire of the private property company to find out more about what older adults and their carers would like to have in terms of services to enable aging in place, using a market survey approach. Therefore, it is not a rigorous academic study using questionnaires that have psychometric properties such as validity and reliability. Furthermore, there is no attempt to claim that the sample is representative of the general population. In fact, we are studying a sub-section of the population that lives in private housing, and hence has a higher income and education. Nevertheless, it is important information, as there is an increasing drive to develop “silver economy”, and from the private sector’s point of view, it is a basic step to elicit potential clients’ wishes in order to develop marketable products. This is the first attempt to explore this topic, and there have not been any similar studies in Hong Kong. Studies relating to aging in place in other countries have been diverse, covering specific areas in depth rather than approaching the topic in a holistic way from the users’ point of view in the real-world setting of private housing in Hong Kong. Due to the high population density in Hong Kong, urban planning and housing have unique features, and it would be difficult to make any international comparisons. Even though the focus of this study is on private housing, and just over 50% of the population lives in public housing, some of the findings may also be applicable to those residing in public housing. Further studies are needed to confirm the generalizability of these findings.

## 5. Conclusions

Aging place is a common feature of many social policies. It is timely to develop pilot models of aging in place that take into account the prerequisites of healthy aging using a sustainable financial model.

## Figures and Tables

**Table 1:** Descriptive characteristics.

	No. (%) with 1017 as the Denominator	
***N*** **= 1017**	Male	Female
Age group (years) **	Total: 414	Total: 603
<50	36 (3.54)	54 (5.31)
50–59	41 (4.03)	100 (9.83)
60–69	87 (8.55)	179 (17.6)
70–79	154 (15.14)	216 (21.24)
80+	96 (9.44)	54 (5.31)
Living arrangement **		
With relatives or a spouse	344 (33.82)	571 (56.15)
Helper only	48 (4.72)	11 (1.08)
Living alone	22 (2.16)	21 (2.06)
**Education ^a^**		
Primary or below	38 (3.75)	66 (6.52)
Up to Secondary	195 (19.25)	291 (28.73)
Tertiary	179 (17.67)	244 (24.09)
Household income (HKD) ^b,^*		
<40 K	188 (18.99)	220 (22.22)
40–70 K	180 (18.18)	305 (30.81)
>70 K	38 (3.84)	59 (5.96)
Carer status ^c,^**		
Yes	160 (15.75)	355 (34.94)
No	253 (24.9)	248 (24.41)
**Self-rated health (combine R1 and R2)**		
Good	274 (26.94)	391 (38.45)
Fair	104 (10.23)	155 (15.24)
Poor	36 (3.54)	57 (5.6)
**No. of chronic diseases**		
0	27 (2.65)	48 (4.72)
1–2	361 (35.5)	505 (49.66)
3+	26 (2.56)	50 (4.92)

^a^ Four missing values were identified. The percentages were calculated with n = 1013. ^b^ Twenty-seven missing values were identified. The percentages were calculated with n = 990. ^c^ One missing value was identified. The percentages were calculated with n = 101. ** Chi square test for analysis between genders *p* < 0.001. * *p* < 0.05.

## Data Availability

The first source of data is from NielsenIQ (NIQ).
